# Post-Activation Performance Enhancement (PAPE) Increases Jumping Power in Elite Female Volleyball Athletes

**DOI:** 10.3390/sports12010022

**Published:** 2024-01-09

**Authors:** Rahel Heynen, Micah Gross, Thomas Betschen, Klaus Hübner

**Affiliations:** Department of Elite Sport, Swiss Federal Institute of Sport Magglingen (SFISM), 2532 Magglingen, Switzerland; micah.gross@baspo.admin.ch (M.G.); thomas.betschen@students.bfh.ch (T.B.); klaus.huebner@baspo.admin.ch (K.H.)

**Keywords:** post-activation performance enhancement (PAPE), muscle power, elite female volleyball players, muscle activation competition, conditioning activity

## Abstract

This study investigated PAPE effects of two conditioning activities (CA) and recovery times on the peak jumping power (PP) of elite female volleyball athletes. Players performed CA separately: three sets of three repetitions of back squats with 85% of 1RM (BS) or one set of five depth drops (DD). PP was measured with countermovement (CMJ) and squat jumps (SJ) before (pre-test) and two minutes (post-test 1) and six hours (post-test 2) after each CA. BS significantly reduced PP at post-test 1 (CMJ and SJ: *p* < 0.04, *d* between −0.36 and −0.28). At post-test 2, following BS, PP for both jump forms was significantly greater than at post-test 1 (*p* < 0.001, *d* between 0.54 and 0.55) and at pre-test (*p* < 0.048, *d* between 0.21 and 0.30). DD increased PP significantly (CMJ and SJ *p* < 0.05, *d* between 0.40 and 0.41) relative to pre-test at post-test 2 (there was no significant difference between pre-test and post-test 1). Comparing BS with DD, there were no significant differences (*p* > 0.05). The greatest PAPE effects were observed six hours after BS. CA are recommended for female athletes to improve jumping performance, but individual responses should be determined prior to use.

## 1. Introduction

Many sports involve sprinting, cutting and jumping actions, which require explosive muscle force production and high muscle power. To enhance power in these situations, athletes usually employ specific strength and power training routines over several weeks or months [1]. Volleyball is a sport with explosive actions (jumping, attacking, blocking, and serving), and jumping power is a key indicator of the required explosive strength ability. For the execution of explosive, sport-specific actions, muscle activation before the match is important [2]. There are several methods of doing so which are known to potentiate muscle force production [3,4]. Post-activation potentiation (PAP) has been used previously to describe an increased muscle force production capacity during an electrically evoked twitch following prior to muscle activation [5]. PAP is a distinct physiological phenomenon with a short time window (seconds to several minutes) in which effects can be observed. These effects can be largely attributed to myosin light chain phosphorylation within type II fibers [6,7]. Post-activation performance enhancement (PAPE), in contrast, differs from PAP in terms of the mechanisms of force enhancement and the time course of effects (few minutes up to 48 h) [7,8]. In general, the longer-lasting PAPE effect can coincide with increased maximal voluntary strength, power, or speed, possibly related to changed muscle temperature, muscle/cellular water content, and/or muscle activation [6,9]. PAPE can be used as a high-intensity conditioning activity (CA), either after no warm-up (physical preparation routine) or a limited/incomplete warm-up, to improve performance in a competition or match [6].

Studies investigating PAPE in sports-related settings have often revealed positive effects. However, due to a multitude of different populations and interventions, generalized conclusions are elusive [3,10,11,12]. Indeed, the cumulative effect sizes (ES) of 179 effects from 36 studies analyzed by Dobbs et al. [12] was trivial (ES = 0.08, 95% CI −0.04 to 0.21, *p* = 0.197). Nonetheless, another meta-analysis was able to isolate the influences of sex, training status, activation exercise, intensity, volume, and rest periods on the magnitude of PAPE, specifically regarding the enhancements to explosive muscle power. Results highlighted the importance of each of these modulators and suggested that the greatest PAPE effects can be expected in male athletes, seven to ten minutes after performing multiple sets of a dynamic, moderate-intensity (60–85% 1RM) CA like the back squat [3]. The study by Masel and Maciejczyk (2022) investigated the effect of PAPE on elite volleyball players. A single set of three repetitions (80% 1RM) of the trap bar deadlift failed to consistently elicit PAPE effects on jump performance in two different jump tests: squat jump (SJ) and countermovement jump (CMJ). The results of this study showed no significant effects, yet high inter-individual differences [13].

Regarding the seemingly smaller PAPE effects found in female athletes, methodology could be as likely a cause as subject sex. Most studies with females have employed CA protocols that are suboptimal with regard to the recommendations given by the same meta-analysis [3]. For example, the study of Sygulla and Fountaine [4] found no significant potentiation (*p* = 0.279) of jumping power five minutes after one set of back squats with a rather heavy load (90% of 1RM) in female college baseball, softball, and volleyball athletes. On the other hand, recent studies with female volleyball players have reported effect sizes of 0.7–0.9, which are in the range of those for males reported by Wilson et al. [3,14,15]. Krčmár et al. [16] compared different PAPE protocols in 14 female athletes (volleyball, track and field, handball, soccer, and cross-fit) on short sprint and vertical jump height: three sets of four repetitions of back squats (85% of 1RM) with an isoinertial load or with different elastic band resistances (20% or 30% of the total resistance bands). The results suggested that all PAPE protocols were able to enhance short sprint performance and vertical jump height. The highest effect sizes were achieved with the 30% resistance band protocol. PAPE effects were also found to be more than twice as large in males than in females [10,12]. Thus, more research on PAPE, specifically in females, is needed to determine which effects can be expected and whether previously formulated recommendations are justified for both sexes.

For a CA to elicit a PAPE effect, parameters such as muscle action type (concentric, eccentric, or isometric muscle activation), exercise volume and intensity, and rest interval between CA and performance must be optimized. A main challenge in this regard is to find the optimal balance between potentiation and fatigue [3,17]. As described in the meta-analysis of Dobbs et al. [12] and in other studies [4,12,14,15,17], the back squat is one of the most commonly used lower-body CA for eliciting PAPE. Furthermore, an intriguing CA was presented by Hilfiker et al., who evaluated the effects of five depth drops from 60 cm with an abrupt landing and 90 degrees of maximal knee flexion in elite athletes from multiple sports [18]. An average improvement of 1.1–2.2% in vertical jumping power and individual enhancements of up to 10% compared to pre-activation were observed one minute post-activation (which was feasibly too early for maximal PAPE). Further evidence exists for PAPE following similar CA, with drop jumps having been shown to increase jumping and sprinting performance [19]. However, both studies only investigated male subjects.

Alongside finding the optimal activation stimulus (i.e., maximal PAPE, minimal fatigue), timing the activation exercises such that time-dependent PAPE effects are present at the moment of the target performance presents a further challenge in research and practical settings [14,20]. Various CA designed to elicit PAPE effects have been shown to enhance the performance beyond a few minutes, six h, and up to 48 h post-activation [10,14,21]. Regarding the potential of CA to improve jumping performance in volleyball players, two time periods are of interest: a few minutes and approximately six hours after activation [21].

Based on the lack of PAPE research on female athletes and the likelihood of back squats and depth drops to elicit PAPE, the aim of this study was to analyze the PAPE effects on vertical jumping power in elite female athletes. In order to contribute doubly to the literature, two different CA (back squats and depth drops) were assessed separately, and the effects were measured at two post-activation time points (two minutes and six hours). In addition, the possibility of there being significant differences between the two different CA was assessed. Another aim of the present study was to analyze the athletes’ individual reactions to CA. Our hypothesis was that the effect of the depth drops CA would be strongest a few minutes after the intervention according to the results of the study from Hilfiker et al. [18], whereas the PAPE effects of back squats would be greatest six hours post-activation [10,14].

## 2. Materials and Methods

**Figure 1:**
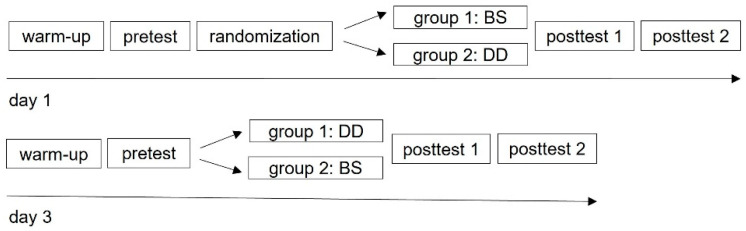
Study design. Procedure of the day one and day three with the tests and condition activities: BS (back squats) and DD (depth drops). Two minutes separated pre-test and post-test 1, whereas six hours separated post-test 1 and post-test 2.

To analyze the PAPE effect magnitudes and timing from two commonly employed CA, this study used a randomized, repeated-measures design (Figure 1). All subjects were exposed to two CA in a randomized order separated by 48 h. Matched pairs of athletes based on CMJ peak power (PP) were split randomly into one of two groups. Group one completed the back squats (BS) CA on the first day (day 1) and the depth drops (DD) CA two days later (day 3), while group two did so in the reverse order.

### 2.1. Subjects

Sixteen healthy elite female volleyball athletes (age = 23.7 ± 6.6 years; height = 180.1 ± 7.8 cm; body mass = 73.1 ± 9.2 kg) volunteered to participate in the study. The initial sample size was reduced because six athletes were unable to complete at least one of the tests due to either absence or physical ailments, leaving ten athletes who performed all tests and interventions (thus, *n* = 10 for all analyses). The participants were members of the Swiss elite national volleyball team and had experience in athletic training including squats (1RM back squat = 99.7 ± 11.4 kg) and plyometrics. The weekly training load including volleyball and strength and conditioning training was 20.3 ± 6.7 h. The study took place in the first week of preparation for the 2021 European championships. Participants were advised to maintain their usual diet, nutritional supplements, and sleep habits during the study. All participants were informed of the benefits and risks of the research. Informed consent was obtained from all participants or their legal guardians, and the study was conducted in accordance with the Declaration of Helsinki and approved by the local Ethics Committee of Canton Bern (Project ID: 2018-00742; 7 June 2018).

### 2.2. Procedures

Interventions and measurements took place in the team’s usual training environment on two separate days, separated by one complete day of normal training. The day before the first test day (day 1), the athletes had a rest day. Athletes’ height (Seca Stadiometer 217) and weight (Seca Robusta 813) were taken at the beginning of day one according to the Swiss Olympic performance testing manual [22]. On both days, after performing their usual pre-game warm-up (dynamic stretching, core stability, sprints, and jumps), which was led by the coach, a vertical jump test (pre-test) was performed, followed directly by the assigned CA. The same vertical jump test was repeated within the next two minutes after completion of the CA (post-test 1), as well as six hours after the CA (post-test 2). Within the first two hours following post-test 1, the team held a technical practice session with low intensity. This was followed by six hours of recovery, including lunch. Just prior to post-test 2, they completed the standard pre-game warm-up again.

The vertical jump test included isolated CMJ followed by SJ. The test procedure has been described in the Swiss Olympic performance testing manual [22]. The jumps were executed with a hip-width stance and hands placed at the hips to eliminate arm swing while jumping. SJ were executed from a static squat position with a knee angle of 90 degrees, which was controlled visually from the side by an investigator. Athletes were requested to jump as high and explosively as possible. Technically incorrect jumps were omitted and repeated, as were jumps whose PP differed from the mean of other trials of the same athlete by 3 W/kg or more (outliers). A force platform (CYCCESS SPSport, Innsbruck, Austria) with accompanying software (Cyccess, Version 2.2.4) provided various kinematic and kinetic jump parameters, of which peak concentric power was retained for assessing performance. The test–retest coefficient of variation (CV) for peak power using the described protocol has been previously determined to be 2.7% (unloaded) for SJ and 2.5% for CMJ.

**Figure 2:**
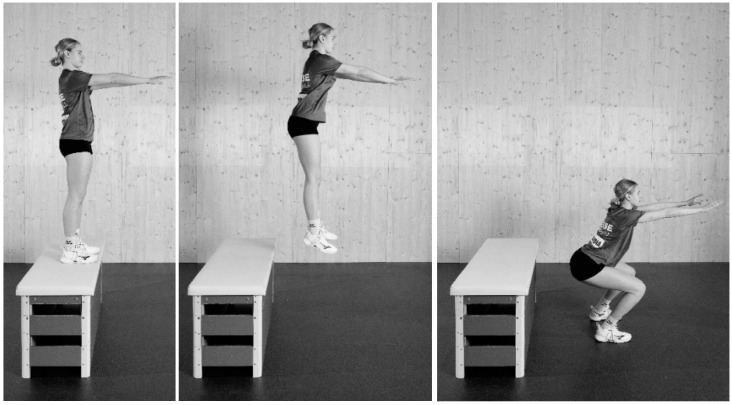
Depth drops (DD) were performed as a set of five repetitions from a 60 cm box and with an active landing and a depth not deeper than 90° of knee flexion.

The DD CA included one set of five depth drops from a 60 cm box. Athletes performed the drops consecutively, separated only by the time required to get back onto the box. The box height was the same for all athletes. Athletes were instructed to perform an abrupt landing, ending in a stable position with 90 degrees of knee flexion according to the study of Hilfiker et al. [18] (Figure 2).

**Figure 3:**
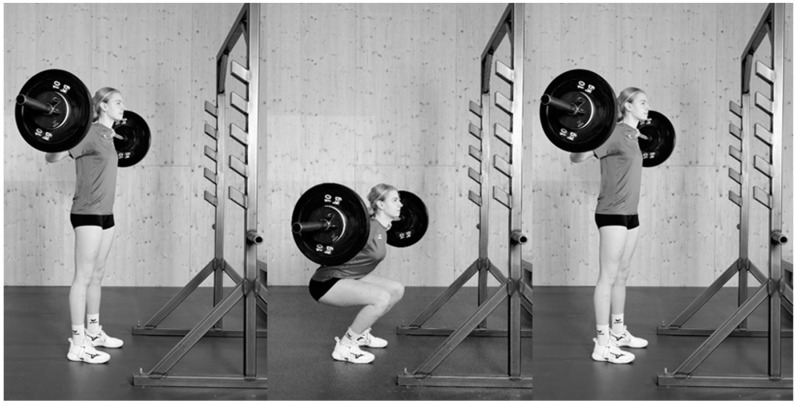
Back squats (BS) were performed as three sets of three repetitions with an estimated 85% 1RM.

The BS CA comprised three sets of three repetitions of BS with 85% of a predicted one-repetition-maximum (1RM) and three minutes of rest between sets. Athletes were instructed to perform the concentric phase as fast as possible. Back squats were performed with feet shoulder width apart and a prescribed depth with thighs horizontal (Figure 3). One week before the study, players five-repetition-maximum load for BS was determined and used for predicting 1RM with the formula of Brzycki [23] and, in turn, the individual loads used for the BS CA. There was no familiarization session because the athletes were familiar with the CA.

In relation to the load between these two CA, there were differences. BS focused on the explosive concentric phase during the squat movement and the muscles under tension were for a longer time. DD had a different stimulus with the explosive contraction active jump landing and shorter time under tension.

### 2.3. Statistical Analyses

For each time point (pre-test, post-test 1, and post-test 2), PP expressed relative to the body mass (W∙kg^−1^) was calculated as the average of the three valid trials of both CMJ and SJ. For these values, body mass taken by the force plate in the same session was used. For each CA and time point, mean ± standard deviation was determined. After having ruled out normal distribution of the dependent variables by means of the Shapiro–Wilk test, the Friedman test was performed on both CMJ and SJ to assess the effects of CA at two post-intervention time points in a repeated-measures fashion. To assess possible main effects of time point, Conover’s post hoc tests with Holm correction were performed. Additionally, BS and DD were compared with each other by performing the Wilcoxon test on individual changes from baseline at each post-intervention time point. Statistical significance was set at *p* < 0.05. Furthermore, effect sizes (*d*) between pairs of time points were calculated as the difference in means expressed as a factor of the pooled standard deviation. Effect sizes were classified according to Cohen [24] as small (≥0.2), medium (≥0.5), or large (≥0.8). Statistics were calculated using Jasp 0.14.1 Software (Jasp, University of Amsterdam, Amsterdam, The Netherlands).

## 3. Results

There was a significant main effect of time point on PP of CMJ and SJ following BS (*p* < 0.001 for both jump forms). Post hoc analyses revealed that BS resulted in significantly reduced PP, with small, negative effect size, at post-test 1 (CMJ: *p* = 0.04, *d* = −0.28; SJ: *p* = 0.03, *d* = −0.36). At post-test 2, following BS, PP for CMJ and SJ were significantly greater than at post-test 1 (*p* < 0.001 and *d* = 0.54–0.55 for both jump forms) and significantly greater than at pre-test (CMJ: *p* = 0.04, *d* = 0.30; SJ: *p* = 0.048, *d* = −0.21).

**Figure 4:**
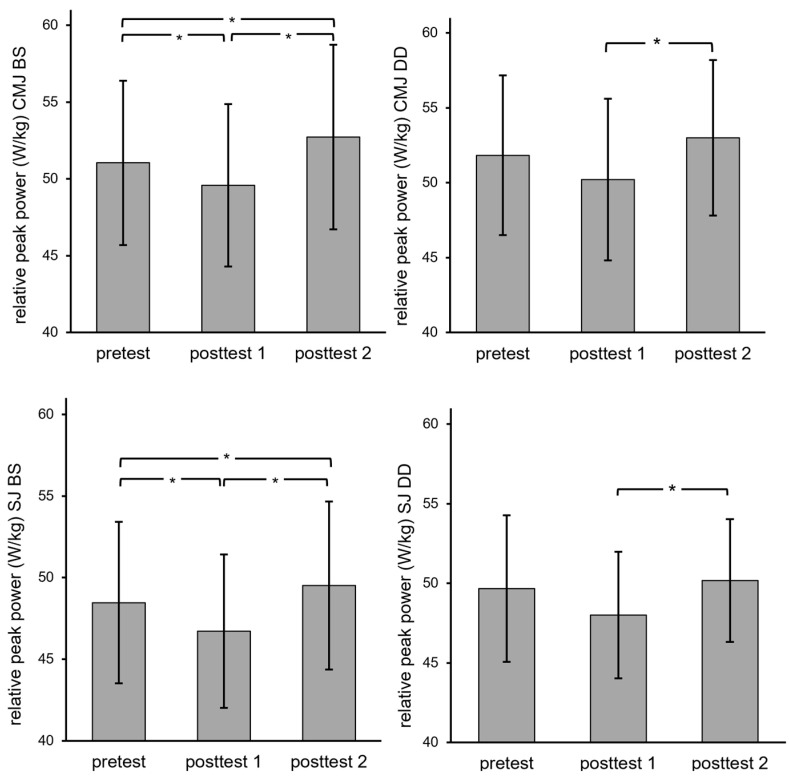
Comparisons of SJ and CMJ peak power (PP) after back squat (BS) or depth drop (DD). Post-test 1: 2 min after conditioning activity (CA). Post-test 2: 6 h after CA. * Significant difference in post hoc analyses *p* < 0.05.

There was a significant main effect of time point on PP of CMJ and SJ following DD (*p* = 0.005 for CMJ, *p* = 0.002 for SJ). Post hoc analyses revealed that PP for CMJ (*p* = 0.01, *d* = 0.40) and SJ (*p* = 0.005, *d* = 0.41) increased significantly between post-test 1 and post-test 2 (with no significant difference between pre-test and post-test 1). However, there was no significant difference in PP between pre-test and post-test 2. These results are displayed in Figure 4.

Comparing BS with DD revealed no significant differences between the two CA in terms of changes from pre-test at either post-test 1 or post-test 2 (*p* from Wilcoxon tests: 0.13–0.83). Individual and group mean data for pre-test along with percent changes from pre-test at both post-CA time points and are displayed in Table 1.

## 4. Discussion

This study investigated PAPE effects in female athletes following two different CA which have been shown to be useful in males. The main findings were that BS diminished PP in the first few minutes but elicited significant positive PAPE effects after six hours. In contrast, DD elicited no significant changes from baseline at either post-intervention time point. No significant differences were found when comparing the two CA in terms of changes from pre-test at either post-test 1 or post-test 2. The results of the study show that the two CA have individual effects on each female athlete. From a physiological point of view, the PAPE could be due to changes in motor unit activation, muscle temperature, or muscle/cellular water content.

The observed enhancements to PP six hours after the BS were for CMJ: 3.0 ± 2.1% and SJ 2.5 ± 1.3% on average (relative to pre-test). Maximal individual effects were 6.6% and 4.5% (Table 1), respectively. This is in line with previous studies that have shown similar effects on PP 6–48 h after performing squats [15,21,25,26,27,28]. Thus, our results suggest that previous conclusions about PAPE effects of BS apply to females as well. However, in contrast to the majority of previous studies [3,10,29] and to our expectation, we observed an impairment to PP in the first few minutes following BS. The study of Sygulla et al. [4] used heavy back squats (one set of three repetitions with 90% of 1RM) as CA in female athletes, and after five minutes rest, static squat jump power was not significantly better. Those authors supposed that one reason could have been the short rest time after muscle activation exercise. That could have been the case in our study as well at pre-test 1, whereas after six hours rest, PP in post-test 2 was significant better. Most of the subjects in previous studies have been male, and we suspect sex may indeed be a factor with regard to effects of this type of intervention. This suspicion seems to be supported by the few previous studies on the topic that were performed with females [14,30].

With regard to DD in the current study, our hypothesis was not confirmed. Contrary to our expectation and to the results of previous studies [14,19], PP was slightly (non-significantly) impaired within the first few minutes after this CA. In the study by Hilfiker et al. [18], an average improvement 1.1–2.2% in PP and individual enhancements of up to 10% compared to pre-activation were observed one minute post-activation. Considering that previous studies showing positive PAPE included both sexes and represented a wide range of jumping abilities [14,18,19,29], neither explosive power ability nor sex stands out as the reason for our unexpected results at two minutes post-CA. Alternatively, fatigue, body composition, passive structures, or anthropological features may have played a role [10,29]. Six hours after DD, PP of CMJ and SJ had returned to the baseline level, with no statistical difference from pre-test. This detriment–recovery pattern may provide evidence that DD were primarily fatiguing and perhaps therefore unsuited for eliciting PAPE effects in the studied cohort. One reason for the lack of significant improvement after DD may be that all participants used the same box height rather than individualized box heights adjusted for body height or strength ability. In comparison to the study of Hilfiker et al. [18], the box height was not adjusted to the athletes’ body size, and significant PAPE effects were found. The standardized box height was also chosen for practical reasons because individualized box heights are unlikely to be available in a competition setting. On the other hand, this study was as practical and close to real-world conditions as possible.

On the other hand, it should not be overlooked that six of the ten athletes improved PP after DD from pre-test to post-test 2 in the CMJ by 7% or more, which is about three times the typical error for that measurement. Moreover, four of those athletes improve and PP in the SJ by 7% or more as well. Thus, an important conclusion based on the current results is the individuality and heterogeneity of responses. In this regard, a more universally beneficial stimulus might have been achieved had we determined the athlete’s individual optimal drop height (e.g., for maximizing eccentric force or rate of force development) and had them perform DD accordingly.

The mechanisms of PAPE are complex. According to the deterministic model introduced by Suchomel et al. [29], athlete characteristics such as sex, training background, strength, muscle characteristic, and neuromuscular factors, as well as characteristics of the intervention itself (exercise, ballistic versus non-ballistic, volume, load, and rest interval), may all play a role in the outcome. Further, the hypothetical interaction between fatigue, potentiation, and performance, as described nicely by Harrison et al. [10], makes clear that not only the effect’s direction and magnitude but also the time course of effects is very individual. In light of this, the fact that all ten athletes displayed positive PAPE responses six hours after one or both CA in the current study (after BS CMJ: 3.0 ± 2.1% and SJ: 2.5 ± 1.3% after DD CMJ: 5.2 ± 4.3% and SJ: 4.3 ± 3.3% average improvement) highlights the potential of these methods in female athlete populations, nonetheless. It appears, however, that female athletes like those in the current study are less likely to respond positively immediately after either type of intervention, perhaps in contrast to males [3]. Sygulla et al. [4] implemented a similar study using BS as a CA with female volleyball players and the results are comparable to those of the present study. Five minutes after CA, the power of squat jumps had decreased. However, the study of Villalon-Gasch et al. [15] analyzed PAPE effects in female volleyball players during a volleyball match. The results showed that three repetitions of back half-squats with 90% of 1RM increased vertical jump power for several minutes during a match. In that study, CMJ was tested during the breaks between the volleyball match (from 8 min after CA to 123 min). The conclusion was that individually appropriate doses of a CA were able to improve vertical jump power.

A possible explanation for differences between sexes is that fatigue may take longer to subside in females, thus delaying a positive balance between potentiation and fatigue. According to research, males have higher motor unit firing frequency along with a greater cross-sectional area of type II fibers [31,32], and the neuromuscular responses of females appear to be delayed, along with a lower level of muscular strength compared to males [33,34]. These may be reasons for the differential effects of PAPE depending on sex. It cannot be determined if sex alone or rather the (moderate) strength level of the current athletes led to the delayed positive effects for responders. A study with amateur female volleyball players used heavy-loaded back squats with velocity loss control as CA in subsequent countermovement jump power. There were no visible significant PAPE effects, despite individual positive responses. The authors suspected that one reason could be the relatively low strength levels of the subjects or genetics [30]. Seitz et al. [7] (a study with rugby players) and Chiu et al. [11] (a study with trained female and male individuals) supposed that individuals with a higher strength level can better profit from PAPE compared to weaker individuals due, in part, to the fact that they fatigue less from the CA. In any case, sex-specific, or, more likely, individualized prescription and timing of interventions seem to be of great importance for practitioners seeking to exploit the benefits of PAPE, particularly in female athletes.

Our research has notable limitations. First, as has been the case for previous studies as well, we took a one-size-fits-all approach (in terms of CA and timing of measurements) to a topic that is recognizably quite individual. Although we were able to show quite clearly how prevalent negative effects in the first few minutes were for the current athlete cohort, maximal effects occurring at some time later may have been missed because measurements were performed at only one additional time point: six hours post-intervention.

As opposed to other studies, for example, Sue et al. [14], Crewther et al. [17], or Hughes et al. [35], we did not perform post-tests between a rest period of three to sixteen minutes after CA. Doobs et al. [12] mentioned that the greater PAPE effects occur after three to seven minutes rest. Second, it is possible that the athletes’ standard pre-match warm-up routine, which they performed prior to pre-tests and which included some jumps and explosive movements, elicited some potentiation and/or fatigue, and we do not know how this interacted with the interventions that followed. Another cofounding factor could be the technical practice session between the two post-test time points. This reflects the tension between optimal research methodology and the field of elite sport. We tried to optimize the study design by taking into account the training routines of the athletes. In addition, we recommend that the box height of the DD be adjusted to the athlete’s height, just as the BS load was adjusted to their strength level. One final limitation was the small sample size in our study. The small sample sizes can be explained by some missing data and, further, the difficulty accessing elite athletes competing in the same sport with a similar athletic level.

For future research, it would be interesting to see if athletes from other sports have similar PAPE effects with DD and BS and if there are differences between the sexes with the same CA. For the proper stimulation between potentiation and fatigue in future research, an effective method for optimizing the CA could be to conduct the BS with velocity loss control until a mean velocity loss of 10% from the first rep of BS is attained, instead of a default of number of repetitions, like in the study of Krzysztofik et al. [30]. Furthermore, investigations on additional physical tests, such as a 50 m sprint test and a change of direction test, may provide further variables on the effects of PAPE.

## 5. Conclusions

In conclusion, directly after both BS and DD, PP was impaired in elite female volleyball athletes (in the majority of individuals and for the group); thus, the investigated methods seem poorly suited for immediate pre-match preparation. On the other hand, six hours was enough time for the group as a whole to recover and for a majority of individuals to enhance PP. Positive PAPE effects were more uniform following BS, presumably because this intervention was individualized, than following DD, which was not individualized. Comparing the two CA revealed no significant differences in terms of group changes from pre-test at either post-test 1 or post-test 2. Based on this research, 3 × 3 back squats with 85% 1RM six hours before the competition is recommended as CA. From a physiological point of view, PAPE activates more motor units, thus enabling better performance in jumping or sprinting during competition. Thus, these CA can be used in different sports to enhance performance. For practical use in other sports, we recommend trying different CA (intensity, volume, and rest time) for each athlete to find the best individual option before using it as muscle activation in competition. It is important to consider the total load during the warm-up and CA and to find the optimal balance between potentiation and fatigue.

## Figures and Tables

**Table 1:** Individual percent changes in peak power (PP) following back squat (BS) and depth drop (DD) in comparison of the pre-test, either 2 min (pos-test 1) or 6 h (post-test 2), following the conditioning activity (CA).

Subject	Jump	Pre-Test PP W/kg	Post-Test 1 BS (%)	Post-Test 2 BS (%)	Post-Test 1 DD (%)	Post-Test 2 DD (%)
1	CMJ	49.8	−4.6	−1.4	−2.6	8.5
	SJ	48.2	−0.9	3.9	−2.0	8.4
2	CMJ	57.7	−3.7	6.6	−3.1	8.4
	SJ	57.0	−4.5	3.5	−0.1	2.4
3	CMJ	41.4	−7.5	3.4	−4.8	8.0
	SJ	40.6	−7.9	4.0	−3.8	7.0
4	CMJ	53.1	−4.2	3.0	−6.4	7.7
	SJ	51.4	−2.6	1.5	−9.4	8.5
5	CMJ	52.0	0.0	1.7	−2.8	9.6
	SJ	50.6	−2.6	1.4	−2.7	8.4
6	CMJ	50.6	1.2	3.1	−4.9	7.4
	SJ	47.5	−2.3	−0.1	0.3	3.0
7	CMJ	46.8	−1.4	4.2	0.2	2.3
	SJ	48.2	−6.2	2.2	−1.8	−1.3
8	CMJ	57.7	−2.6	4.2	−0.4	3.2
	SJ	53.7	−2.4	3.9	−2.3	2.3
9	CMJ	50.8	−2.2	4.5	3.1	2.1
	SJ	46.2	−3.3	2.2	0.9	3.4
10	CMJ	58.5	−5.9	0.5	2.5	−4.7
	SJ	53.3	−6.6	2.7	−2.7	1.0
CMJ mean ± SD		51.8 ± 5.1	−3.1 ± 2.5	3.0 ± 2.1	−1.9 ± 3.0	5.2 ± 4.3
SJ mean ± SD		49.7 ± 4.4	−3.9 ± 2.2	2.5 ± 1.3	−2.4 ± 2.7	4.3 ± 3.3

## Data Availability

Data from this study are unavailable due to ethical restrictions.
